# Assessment of cardiometabolic risk in children in population studies: underpinning developmental origins of health and disease mother–offspring cohort studies

**DOI:** 10.1017/jns.2014.69

**Published:** 2015-04-10

**Authors:** R.-C. Huang, Susan L. Prescott, Keith M. Godfrey, Elizabeth A. Davis

**Affiliations:** 1 Telethon Kids Institute, University of Western Australia, Roberts Road, Subiaco, WA, Australia; 2 ‘In-FLAME’ the International Inflammation Network, World Universities Network (WUN); 3 Department of Endocrinology, Princess Margaret Hospital, Roberts Road, Subiaco, WA, Australia; 4 School of Paediatrics and Child Health Research, University of Western Australia, Roberts Road, Subiaco, Australia; 5 MRC Lifecourse Epidemiology Unit and NIHR Southampton Biomedical Research Centre, University of Southampton and University Hospital Southampton NHS Foundation Trust, Southampton, UK

**Keywords:** Paediatric health, Metabolic disease, Cardiovascular risk, Population studies, DOHaD, developmental origins of health and disease, DXA, dual-energy X-ray absorptiometry, IMT, intima media thickness, NAFLD, non-alcoholic fatty liver disease, PWV, pulse wave velocity, TNFR, TNF receptor

## Abstract

Pregnancy and birth cohorts have been utilised extensively to investigate the developmental origins of health and disease, particularly in relation to understanding the aetiology of obesity and related cardiometabolic disorders. Birth and pregnancy cohorts have been utilised extensively to investigate this area of research. The aim of the present review was twofold: first to outline the necessity of measuring cardiometabolic risk in children; and second to outline how it can be assessed. The major outcomes thought to have an important developmental component are CVD, insulin resistance and related metabolic outcomes. Conditions such as the metabolic syndrome, type 2 diabetes and CHD all tend to have peak prevalence in middle-aged and older individuals but assessments of cardiometabolic risk in childhood and adolescence are important to define early causal factors and characterise preventive measures. Typically, researchers investigating prospective cohort studies have relied on the thesis that cardiovascular risk factors, such as dyslipidaemia, hypertension and obesity, track from childhood into adult life. The present review summarises some of the evidence that these factors, when measured in childhood, may be of value in assessing the risk of adult cardiometabolic disease, and as such proceeds to describe some of the methods for assessing cardiometabolic risk in children.

## Why is assessing cardiovascular risk in childhood important for developmental origins research?

**Fig. 1.**
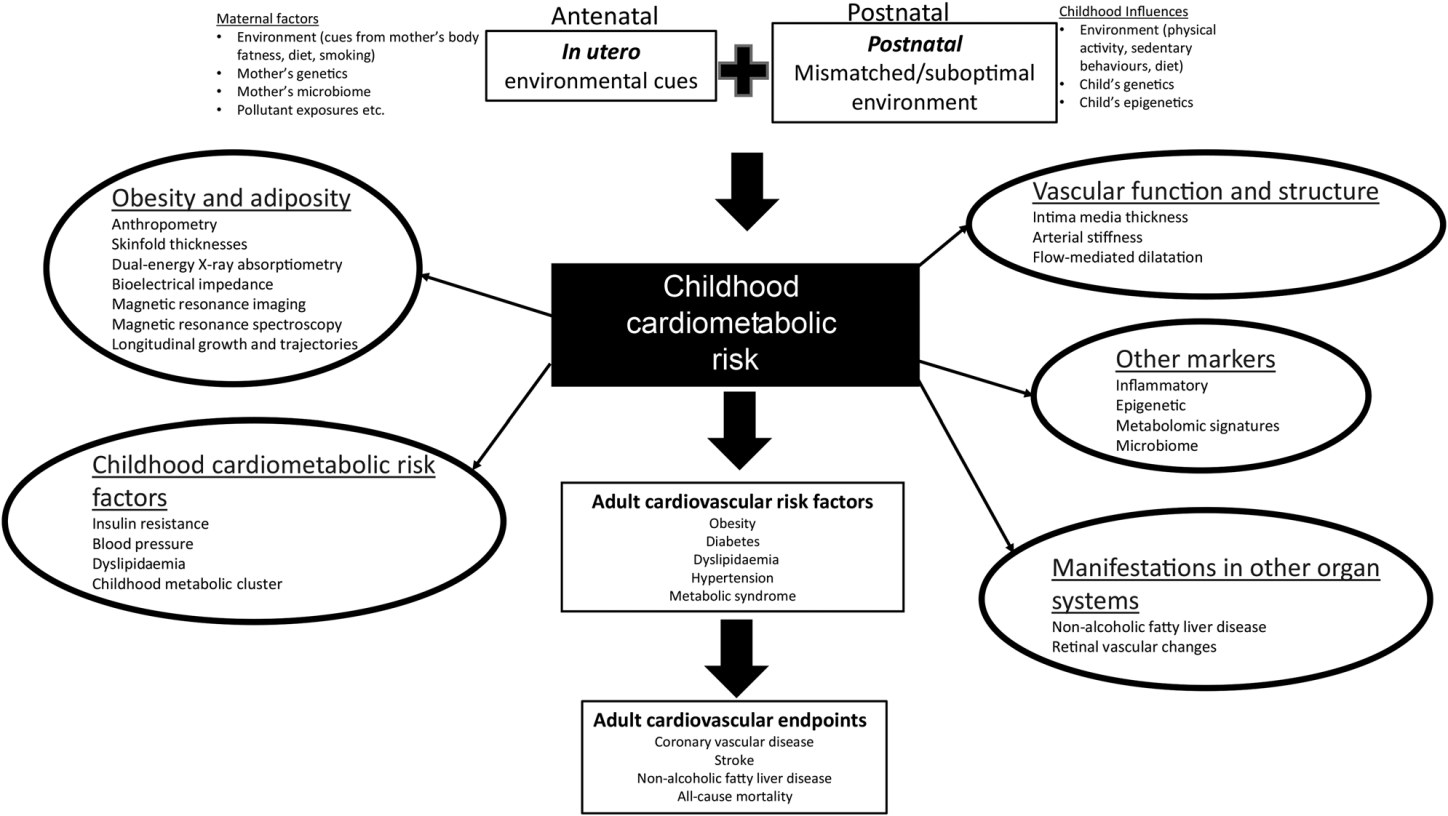
Purported pathways involved in developmental origins of health and disease concepts. Cardiometabolic risk can be both an outcome and also a mediator towards ultimate CVD.

A geographical correlation between infant and later CVD mortality provided some of the first evidence that adverse conditions during development could have latent and long-term effects on diseases that were previously considered to have their origins during adulthood^(^^1^^)^. Following earlier speculation that this may reflect maternal/infant nutrition at critical stages of development, David Barker's team went on to report evidence that lower birth weight was an independent risk factor for later CHD and the metabolic syndrome. In this context, low birth weight represents a tangible, albeit multifactorial, reflection of a suboptimal *in utero* environment. The resulting developmental origins of health and disease (DOHaD) paradigm proposed that a range of metabolic, immunological and physiological adaptations to suboptimal antenatal conditions acts in concert with postnatal conditions to modify subsequent disease risk (Fig. 1)^(^^2^^)^.

The quality of the *in utero* environment is influenced by broad-ranging maternal environmental factors, maternal general health and fixed genetic variability. Experimentally, maternal environmental influences which have been studied include nutritional status (in the periconceptional period^(^^3^^,^^4^^)^ and during pregnancy^(^^5^^,^^6^^)^), physical activity, pharmacological agents (prescribed and illicit), patterns of microbial diversity, smoking and pollutants. Some of these have been shown to have epigenetic effects on diverse aspects of fetal development. A similar set of influences operates, variably, in the postnatal environment, also interacting with the genetic and epigenetic processes to affect developmental processes. Within the schema shown in Fig. 1 cardiometabolic risk is important as both an outcome measure and as a mediator/predictor of future health and well-being.

The growing global burden of obesity is now affecting all regions of the world, and associated cardiometabolic disorders are among the greatest threats to human health. To address this public health crisis, it is important to understand why obesity has developed so rapidly in such a short space of time. Obese women and those who develop gestational diabetes are more likely to have offspring who themselves are obese^(^^7^^)^ and have an increased risk of metabolic disease^(^^8^^,^^9^^)^. This suggests that early life effects (potentially through obesity and hyperglycaemia during pregnancy) could be an important factor in the rising obesity rates being observed worldwide.

Studies of short-lived animals, which can be readily undertaken across the entire lifespan (from before conception to the disease end point of interest), have consistently provided support for the DOHaD concept. This is far more challenging in human studies which require long-term longitudinal follow-up. There are further differences between animal and human studies. Most of the animal models rely on controlled nutritional interventions^(^^10^^,^^11^^)^ which have provided strong evidence of a causal relationship between early-life exposure and metabolic risk in later life. Early-life exposures in humans are much more complex and multifactorial, with exposures such as smoking, drug exposure, stress and toxins at play. Despite these limitations, it is critical that the observed effects on fetal programming are replicated in human studies. Further, it is important to rigorously ascertain whether interventions aimed at favourably altering fetal programming are effective in humans.

Human DOHaD research has been conducted in many prospective mother–offspring cohorts and in a broad range of early-life intervention studies. Most current prospective population studies^(^^12^^–^^14^^)^ and almost all intervention studies^(^^15^^–^^18^^)^ involve offspring who have not yet reached old age, or even middle age. As a consequence, they often do not as yet have definitive CVD endpoints (such as CHD and stroke). So far, the reported outcomes from these prospective human studies are less definitive ‘risk’-associated parameters measured from childhood, adolescence or young adult life (see below). These prospective mother–offspring studies *per se* will not be able to establish direct cause-and-effect relationships between specific exposures and clinical outcomes. Nevertheless they (in combination with animal models and human randomised controlled trials) will play a key role in building the overall picture of the role of DOHaD in human populations.

The utility of cardiovascular risk markers depends on evidence that these ‘track’ from adolescent/childhood through to adult life and, by extrapolation, that those with elevated risk measures earlier in life are also more likely to suffer CVD later in life^(^^19^^–^^23^^)^. Specifically, many studies demonstrate that a spectrum of cardiovascular risk factors including hypertension^(^^19^^)^, dyslipidaemia^(^^20^^)^, obesity^(^^21^^)^ and the metabolic syndrome^(^^22^^,^^23^^)^ track from childhood into adulthood. For example, in the Bogalusa Heart Study, twice the expected number of subjects whose blood pressure levels were in the highest quintile of blood pressure in childhood remained in the highest part of the distribution 15 years later^(^^19^^)^. Similarly, overweight 2- to 5-year-olds in the Bogalusa study were more than four times more likely to become overweight adults, compared with children classified with BMI less than the 50th centile^(^^21^^)^. In the Fels study, a child or adolescent with a high BMI percentile for age remained at high risk of being overweight or obese at 35 years of age. Interestingly, this risk increased in magnitude with increasing age^(^^24^^)^. A systematic review has shown that all included studies consistently report an increased risk of overweight and obese youth becoming overweight adults^(^^25^^)^.

Several key postnatal factors, in particular postnatal weight gain, have been shown to predict cardiovascular risk, independent of birth size^(^^26^^)^ and early childhood obesity may be on the pathway between early-life factors and cardiovascular outcomes. As such, childhood obesity and insulin resistance can be utilised as both determinants of cardiovascular risk and/or as outcomes in epidemiological models. Clearly, childhood obesity is a major health issue in its own right with a broad range of immediate health risks, in addition to the long-term risk of CVD and many non-communicable diseases^(^^27^^)^.

## Assessing obesity and adiposity in children

Alongside the virtually universal measures of BMI, prospective cohort studies often include other anthropometric measures including waist circumference, skinfold thicknesses, and sometimes measurements of body composition from DOHaD (dual-energy X-ray absorptiometry; DXA) and bioimpedance methodologies.

### Other methods of assessing adiposity in childhood

In infants and children BMI is generally a less predictive measure of overall adiposity than in adults, and methods for assessing adiposity in cohort studies have recently been reviewed^(^^28^^)^. More refined methods of measuring adiposity and body composition in childhood may have utility in dissecting the role of the contribution of DOHaD mechanisms to ultimate cardiovascular risk. A long-recognised and consistent finding is that low birth weight followed by postnatal weight gain is associated with a central distribution of adiposity, increased percentage of body fat and increased skin folds^(^^29^^,^^30^^)^. High birth weight is also associated with later obesity risk. This suggests that both impaired and excessive growth *in utero* have effects on programming for obesity. Therefore, measuring body composition may provide greater sensitivity to understanding the early programming of obesity. The most commonly used of these techniques include DXA^(^^31^^)^, bioelectrical impedance^(^^32^^)^, air displacement plethysmography, MRI and magnetic resonance spectroscopy (MRS). There is emerging evidence that ectopic fat deposits such as in the renal sinus, myocardial region and peripancreatic regions are best quantified using MRI^(^^33^^)^. Bioelectric impedance is portable and relatively inexpensive, but not as accurate as DXA or MRI. Bioelectric impedance is particularly prone to inaccuracies with changes in body water:adipose ratio, as could occur with illness and dehydration^(^^32^^)^. When employing techniques such as MRI, MRS and DXA in large-scale longitudinal studies, considerations of cost and time commitment to the participants are necessary, particularly if these measurements are to be repeated at several follow-ups. DXA cannot distinguish between visceral and subcutaneous fat.

### Cross-sectional assessment of anthropometric measures

In the literature on adults, there has been much debate about the anthropometric measures that best predict cardiovascular risk in cross-sectional and longitudinal studies. It is generally agreed that measures of central adiposity and abdominal visceral fat deposition, such as waist:hip ratio and waist circumference^(^^34^^)^, are likely to be superior to BMI, at least in adults.

However, the role of these measures in childhood is less definitive. Rather, there is some evidence that measures of central obesity in children are not more predictive of cardiometabolic risk than BMI *Z* score^(^^35^^,^^36^^)^. In contrast to adults, waist:hip ratio in children does not predict blood lipids, blood pressure and traditional cardiovascular risk factors. However, there are some metabolic risk markers, namely fasting TAG and homeostatic model assessment of insulin resistance (HOMA-IR), that do appear to be associated with anthropometric measures such as BMI or waist circumference^(^^30^^)^. Furthermore, cholesterol and LDL are inversely associated with height. In childhood, there may be no ‘best’ cross-sectional measure of cardiovascular risk, and the choice of optimal cross-sectional anthropometric measure should depend on the research question being asked.

### Longitudinal assessment of anthropometric measures

Fortunately most birth cohorts have repeat measures of anthropometry which provide the opportunity for longitudinal statistical modelling to be applied to obesity measures. Longitudinal measures may have more value than cross-sectional measures in answering DOHaD-related questions. The present review is not intended as a comprehensive review of statistical longitudinal methods or of relative efficiencies of each method, but considers how these measures may be most relevant to investigating DOHaD phenomena. Suffice to say, there are many techniques for investigating longitudinal measures including linear mixed-effects model, linear mixed-effects model with skew-t random errors, semi-parametric linear mixed models, latent class models and non-linear mixed-effects modelling. Careful selection of the most appropriate statistical tool to answer each different life-course question is critical.

Pathways to childhood obesity are likely to be heterogeneous, and under the influence of a number of maternal and childhood factors acting at different time points (Fig. 1). Longitudinal statistical techniques, particularly those that identify different patterns of growth are useful^(^^37^^–^^39^^)^ particularly as they assume and identify different, and potentially causal, pathways to obesity. For example, there is evidence that childhood obesity can occur through DOHaD effects related to large-for-gestational-age neonates who are exposed to the *in utero* effects of gestational diabetes and maternal obesity^(^^9^^)^. By contrast, childhood obesity can also be driven by starvation *in utero* as occurred to fetuses exposed during the Dutch Hunger Winter^(^^40^^)^.

## Assessing cardiometabolic risk in children

Obesity does not usually occur in isolation, but generally occurs within a cluster of abnormalities that includes hypertension, dyslipidaemia and insulin resistance. There are some points to note, specific to children, when interpreting these individual risk factors. *Z* scores of blood pressure specific for age, sex and height of the child are most appropriate for evaluation of high blood pressure in childhood^(^^41^^)^. Likewise, age, sex and puberty all affect fasting total cholesterol, LDL, HDL, TAG and insulin levels through childhood^(^^42^^)^.

The co-occurrence of these risk factors, ‘Syndrome X’, is a phenomenon identified by Reaven in 1988, and subsequently recognised by various different names, most commonly now as the metabolic syndrome^(^^43^^)^. Defining the metabolic syndrome in children is problematic. In adults, the definition is based upon arbitrary cut-offs with three main consensus definitions (National Cholesterol Education Program (NCEP), WHO and the International Diabetes Federation (IDF))^(^^44^^,^^45^^)^. Consensus definitions all vary slightly in terms of the cut-off limits used, and it is important to emphasise that these apply a bimodal approach to risk factors that generally have continuous relationships with disease across the range. They also vary with ethnicity, and adult definitions are not appropriate to translate to studies of children. At present, in children, there is no consensus around definitions of the metabolic syndrome. As an illustration of this controversy, in 2008, in excess of forty unique paediatric definitions of the metabolic syndrome in children had been used in the literature^(^^46^^)^. Two of the more recent major definitions were compared with different groups being identified in the same population. The IDF metabolic syndrome criterion represents a more stringent definition that was adapted from the NCEP definition^(^^47^^)^.

To overcome some of these issues of definition in children, one approach is to use ‘data-driven’ methods to avoid use of arbitrary cut-offs. This involves methods that can identify natural groupings within a population. For example, cluster analysis is a data-driven method that identifies groups maximising within-group similarities and maximising between-group differences using a variety of statistical algorithms^(^^48^^)^.

**Fig. 2.**
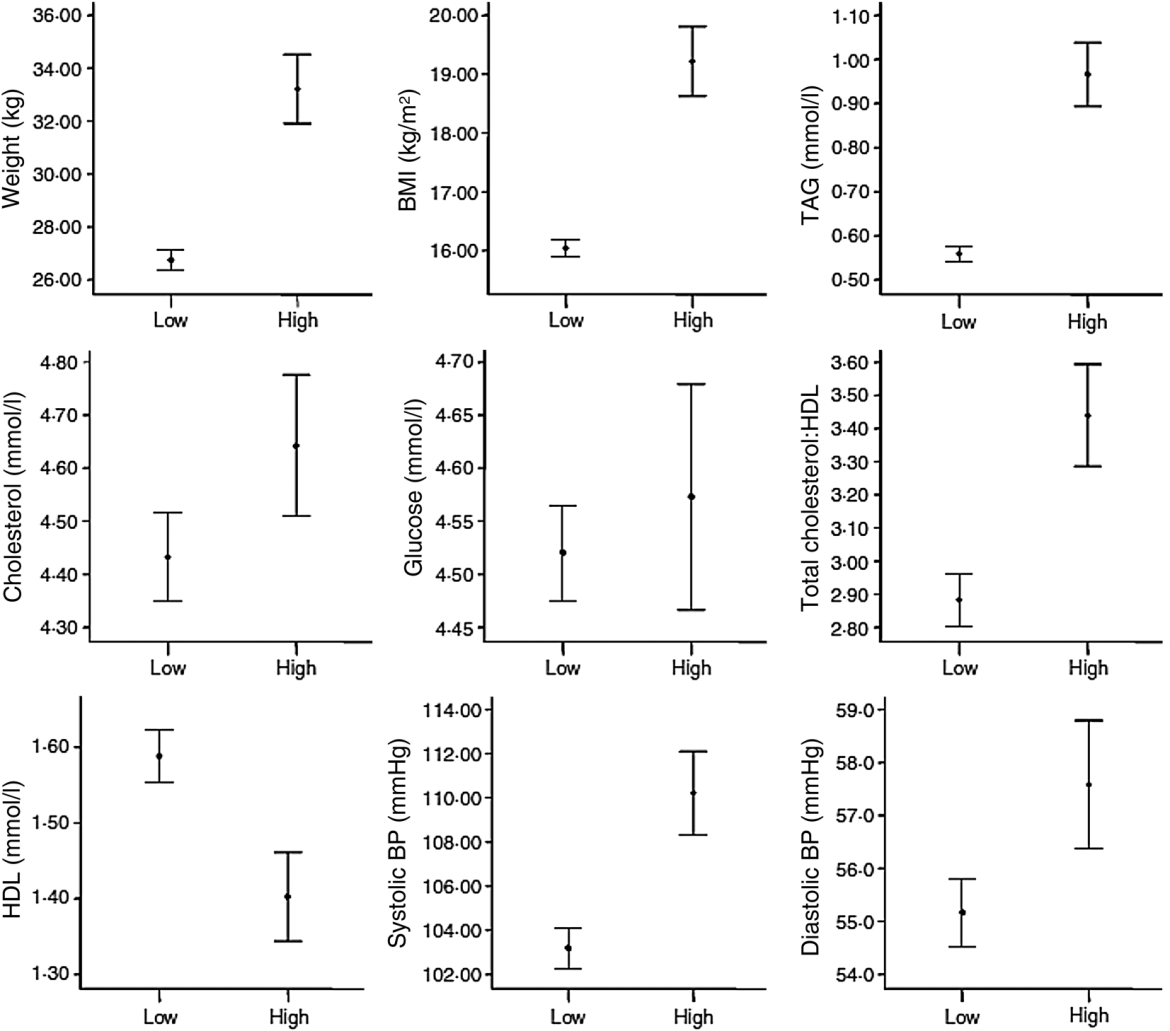
95% CI for parameters related to high-risk cluster at age 8 years. Most of the 95% CI are very divergent and do not overlap. The x axis shows those in high- and low-risk clusters^(^^14^^)^. BP, blood pressure.

This approach was used in the West Australian Pregnancy Cohort (Raine) Study. Specifically, cluster analysis was undertaken using continuous variables (BMI, systolic blood pressure, fasting serum TAG and HOMA-IR) to identify a group of children with features similar to the metabolic syndrome. At ages 8, 14 and 17 years, 29%^(^^14^^)^, 24%^(^^49^^)^ and 19% of the population, respectively, were identified as in the ‘high metabolic risk’ cluster. These groups were highly divergent for traditional risk factors (Fig. 2), and included a substantially larger proportion of the population than would have been defined by conventional definitions. This gives greater power to analyse associations with these metabolic cluster groups, as compared with groups defined using metabolic syndrome definitions. Utilising this cluster technique, the U-shaped relationship with birth weight was shown in a contemporary Western population^(^^14^^)^. Children who originated in the lowest and highest birth-weight quintiles had significantly greater odds of being classified at high metabolic risk by middle childhood compared with those in the middle nadir birth-weight quintile.

Although data-driven cluster analysis can be used within a specified population, it does not necessarily define cut-offs that can be translated to another population. For cross-cohort comparisons, a consensus definition based on cut-offs is still required. As such, there remains a need for expert committees to define consensus statements.

Currently there is insufficient longitudinal data to know what cut-offs predict future disease. Nevertheless, despite the varying definitions, overall stability of risk factor clustering is seen from childhood into adult life^(^^50^^)^. Therefore, clustering of risk factors in childhood is predictive of risk of development of the metabolic syndrome in subsequent adult life

## Other cardiovascular risk markers in children

Detecting risk of CVD in children, before the expression of overt cardiovascular endpoints, has been achieved via other methods. These methods include assessments of vascular structure and function, inflammatory and epigenetic biomarkers, non-alcoholic fatty liver disease (NAFLD) and the retinal vasculature. In making a decision about which of these diverse methods are best employed in any particular study, two factors should be considered. The first is that methods may potentially target different pathways in the evolution and the eventual development of CVD. Ideally the technique(s) chosen should link to the research question being asked. The second consideration is a practical one. Some methods are more time intensive, expensive and demanding of greater expertise, and need to be justified in terms of the greater burdens placed upon study participants and resources.

### Markers of vascular structure and function

Vascular structure and function can be measured by intima media thickness (IMT), pulse wave velocity (PWV) and flow-mediated dilatation (FMD).

Atherosclerosis is present in youth, beginning as deposits of cholesterol and its esters in the endothelial wall. The Pathobiological Determinants of Atherosclerosis in Youth (PDAY) study performed autopsies on 3000 individuals aged 15 to 34 years dying of unrelated causes^(^^51^^,^^52^^)^. Evidence of atherosclerosis was directly observed even at these relatively young ages, in the form of fatty streaks and narrowing of coronary vessels. This has driven the development of non-invasive techniques that can be used to detect evidence of early atherosclerosis in childhood and later cardiovascular risk. These are indirect measures of subclinical atherosclerosis and interpretation of these results in children needs to be undertaken carefully.

### Intima media thickness

One method, established in children, for assessing early morphological changes in the vessel wall is measurement of aortic and carotid IMT. This technique has confirmed that traditional cardiovascular risk factors (such as obesity, diabetes, hypercholesterolaemia and hypertension) in childhood are associated with the formation of early atherosclerotic lesions^(^^53^^–^^55^^)^. Jarvisalo *et al.*^(^^53^^)^ showed that, at an average age of 11 years, children with type 1 diabetes and hypercholesterolaemia had higher IMT compared with a control group. Children with hypertension (at a mean age of 13·9 years) also had significantly greater carotid IMT thickness than unaffected children^(^^54^^)^. Finally, overweight children have been shown to have significantly increased carotid IMT, even for mild to moderate degrees of obesity^(^^55^^)^. Lower maternal energy intake during pregnancy has also been shown to be associated with increased carotid IMT in 9-year-old children^(^^56^^)^.

### Arterial stiffness

Arterial compliance or stiffness is another non-invasive measure of later cardiovascular risk. Three non-invasive methods of measuring arterial stiffness are used: (1) measuring PWV; (2) relating change in diameter (or area) of an artery to distending flow; and (3) assessing arterial pressure waveforms. Using these measures, cardiovascular risk can be objectively and non-invasively quantified using applanation tonometry (Sphygmocor) which measures arterial stiffness. Measures of PWV and augmentation index predict cardiovascular events and mortality independent of other traditional risk factors in adults. Increased arterial stiffness has been found in high-risk groups of children such as those with obesity^(^^57^^)^, type 2 diabetes^(^^58^^)^ and familial hypercholesterolaemia^(^^59^^)^. In the Raine study, we have seen that the high ‘metabolic risk cluster’ participants have higher PWV in both sexes. In males, those in the higher metabolic cluster had higher augmentation index (derived from the arterial pressure waveform) in males, but not in females^(^^60^^)^.

Methods of FMD and PWV measure dynamic changes in the vasculature. Therefore, experimental conditions need to be controlled for effects such as exposure to cigarette smoke and for menstrual cycle phase for adolescent girls. Notably, IMT measures a structural change and is not sensitive to these immediate influences.

### Inflammatory and epigenetic biomarkers

C-reactive protein (CRP) is the most studied of the inflammatory markers in relation to cardiovascular risk. It is a non-specific measure of systemic inflammation, and adult studies show that elevated CRP is associated with an increased risk of subsequent cardiovascular risk and all-cause mortality^(^^61^^,^^62^^)^. This has also been seen in children, which shows that elevated CRP levels are associated with increased metabolic risk^(^^63^^)^, arterial changes in healthy children^(^^64^^)^ and eventual CVD^(^^65^^)^. Mendelian randomisation approaches suggest that these associations may not be causal^(^^66^^)^.

Adipokines are cytokines produced by adipose tissue and might provide a mechanistic link between obesity and CVD. Adipokines include adiponectin and leptin. Plasma leptin concentrations correlate with body fat and BMI and may play a role in the aetiology of hyperinsulinaemia and the insulin resistance syndrome^(^^67^^)^. In adolescents, plasma leptin has been associated with insulin resistance^(^^68^^)^.

Other circulating cytokines associated with cardiovascular risk in childhood and adolescence include IL-18, soluble TNF receptors (TNFR) and interferon-*γ*. In adults, high levels of plasma IL-18 are associated with central obesity^(^^69^^)^, the metabolic syndrome^(^^70^^,^^71^^)^ and CVD^(^^72^^)^. In adolescents, IL-18 has been associated with BMI and insulin resistance^(^^73^^)^.

The effects of TNF-*α* are mediated by two specific receptors, a 55 kDa protein (TNFR1) and a 75 kDa protein (TNFR2)^(^^74^^)^. Soluble forms of both receptors are detectable in plasma and have been used as proxies for TNF-*α* activity^(^^75^^)^. Elevated plasma levels of TNFR have been associated with childhood obesity^(^^76^^,^^77^^)^ and cardiovascular events^(^^78^^,^^79^^)^.

Interferon-*γ*-induced protein of 10 kDa (IP-10) is a pro-inflammatory chemokine generated by monocytes to promote the recruitment of lymphocytes and monocytes to sites of inflammation. It is expressed in human atherosclerotic plaques^(^^80^^)^ and plasma levels have been correlated with waist circumference and BMI in adolescents^(^^73^^)^.

Recent studies suggest that epigenetic marks in proxy tissues may reflect mechanistic pathways linking the early environment with later adiposity and differential risk of CVD^(^^81^^,^^82^^)^. As yet there are few data for more direct measures of cardiovascular risk, but it is of note that there is now evidence that some differentially methylated CpG sites are temporally stable between the ages of 5–7 and 14 years^(^^82^^)^.

### Non-alcoholic fatty liver disease

As discussed above, obesity is a spectrum from isolated overweight to the full cluster of co-morbidities, but typically seen in the metabolic syndrome. Similarly, the metabolic syndrome is also associated with other conditions such as NAFLD, the most prevalent chronic liver condition worldwide. The development of NAFLD is associated with key components of the metabolic syndrome. Individuals with NAFLD typically have greater levels of BMI *Z* score, waist circumference, HOMA and systolic blood pressure^(^^83^^)^. An evolving area of interest is that of the microbiome. The effects of diet on metabolic liver disease may be mediated, at least in part, by the microbiome^(^^84^^)^. Ongoing and future mother–birth cohort studies will be analysing the microbiome at different time points.

Population studies that are large scale and performed on healthy participants will have ethical constraints for obtaining ‘gold standard’ liver histology by liver biopsy. Therefore, non-invasive methods for assessing NAFLD are utilised, and MRI is now accepted as producing reliable assessments of liver fat. Many population studies have used liver ultrasound. Assessment by ultrasound may potentially introduce false negatives, but is feasible in population studies^(^^60^^,^^85^^)^.

## Conclusion

Assessing cardiometabolic risk in children is important in understanding developmental programming and how these pathways may be addressed for disease prevention. These cardiometabolic risk factors can either be predictors or outcomes in analyses. Current data suggest that early measures of cardiometabolic risk do track into adulthood and predict cardiovascular outcomes. When assessing the role of adiposity in children, longitudinal statistical techniques and measures of body composition are likely to be useful. Data-driven techniques, such as cluster analysis, should be considered when assessing the metabolic risk markers in children. The development of more sophisticated cardiovascular risk markers in children is constantly evolving. It is important that there is considered use of these techniques in the context of the pathogenic pathways being examined. These approaches will add valuable knowledge to the expanding frontier of developmental medicine (DOHaD), which is ultimately the most logical target for preventing disease and curtailing the rising burden of CVD and other non-communicable disorders.
